# Supplementary material for: Content-rich biological network constructed by mining PubMed abstracts
Source: BMC Bioinformatics. 2004 Oct 8;5:147. doi: 10.1186/1471-2105-5-147 (PMC528731; doi:10.1186/1471-2105-5-147)
Supplement: Additional File 2 — The original results of the above study (non-essential files are deleted to keep the file size under the limit set by BMC bioinformatics). [file 1471-2105-5-147-S2.bz2 › chilibotAdditionalFile2/dip20/553ID7592797E10913/html/IFNAR1_IFNA8.html]

 


 **IFNAR1** and **IFNA8** 
  
Found 13 abstracts in PubMed, retrieved 13.  
 

 What does Google say? 
 PDF only 
| .edu only 

---

**Interactive relationship** (e.g. stimulation, inhibition, etc)

**Non-interactive relationship** (e.g. studied together, co-existance, homology, etc.)

- As with a natural human buffy coat interferon alpha  [ **IFNAR1** ]  preparation, three subtypes LeIF B  [ **IFNA8** ] , C and D showed considerable activity on RK 13 rabbit cells, but two LeIF D and F also showed some activity on mouse L 929 cells.  Ref: 6172550 J Gen Virol, 1981
